# Microbial Community Dynamics and Metabolome Changes During Spontaneous Fermentation of Northeast Sauerkraut From Different Households

**DOI:** 10.3389/fmicb.2020.01878

**Published:** 2020-08-05

**Authors:** Xiaozhe Yang, Wenzhong Hu, Zhilong Xiu, Aili Jiang, Xiangyan Yang, Gaowa Saren, Yaru Ji, Yuge Guan, Ke Feng

**Affiliations:** ^1^School of Bioengineering, Dalian University of Technology, Dalian, China; ^2^College of Life Science, Dalian Minzu University, Dalian, China; ^3^Key Laboratory of Biotechnology and Bioresources Utilization, Ministry of Education, Dalian, China

**Keywords:** homemade northeast sauerkraut, microbiota, metabolome, flavor, correlation

## Abstract

Sauerkraut, one of the most popular traditional fermented vegetable foods in northern China, has been widely consumed for thousands of years. In this study, the physicochemical characteristics, microbial composition and succession, and metabolome profile were elucidated during the fermentation of traditional northeast sauerkraut sampled from different households. The microbial community structure as determined by high-throughput sequencing (HTS) technology demonstrated that Firmicutes and Proteobacteria were the predominant phyla and *Weissella* was the most abundant genus in all samples. Except for *Weissella*, higher relative abundance of *Clostridium* was observed in #1 sauerkraut, *Clostridium* and *Enterobacter* in #2 sauerkraut, and *Lactobacillus* in #3 sauerkraut, respectively. Meanwhile, Principal component analysis (PCA) revealed significant variances in the volatilome profile among different homemade sauerkraut. Acids and lactones were dominant in the #1 sauerkraut. The #2 sauerkraut had significantly higher contents of alcohols, aldehydes, esters, sulfides, and free amino acids (FAAs). In comparison, higher contents of terpenes and nitriles were found in the #3 sauerkraut. Furthermore, the potential correlations between the microbiota and volatilome profile were explored based on Spearman’s correlation analysis. Positive correlations were found between *Clostridium*, *Enterobacter*, *Lactobacillus*, *Leuconostoc*, *Weissella* and most volatile compounds. *Pseudomonas*, *Chloroplast*, *Rhizobium*, *Aureimonas*, and *Sphingomonas* were negatively correlated with volatile compounds in sauerkraut. This study provided a comprehensive picture of the dynamics of microbiota and metabolites profile during the fermentation of different homemade northeast sauerkraut. The elucidation of correlation between microbiota and volatile compounds is helpful for guiding future improvement of the fermentation process and manufacturing high-quality sauerkraut.

## Introduction

Since ancient times, fermented foods are characterized by extended shelf lives and improved organoleptic properties. Through fermentation by microorganisms, products exhibit special microbiota, flavor, and nutritional properties. Chinese fermented vegetables are popular foods that date back to the 6000 bc (Azam et al., 2017). Fermented vegetables are usually prepared under non-sterile fermentation conditions, and a number of microorganisms participate in the fermentation process. Sauerkraut is one of the well-known traditional fermented vegetable in northern China and has been widely consumed for hundreds of years (Zhou et al., 2018). Generally, cabbages are pretreated and immersed in a 0.5–3.5% salt solution, and then left at an ambient temperature (18–20°C) for 1 month. Traditional northeast sauerkraut was fermented based on naturally occurring microorganisms present on the raw material, such as lactic acid bacteria (LAB), Enterobacteriaceae, Pseudomonadaceae, and yeasts (Wouters et al., 2013). This complex fermentation process can improve the nutrition and enrich the flavor of sauerkraut.

During fermentation, microbes play a vital role in the formation of flavor and enhancement of nutritive value. In particular, LAB, the major bacterial community, possess enzymes capable of hydrolyzing many food constituents, including carbohydrates, proteins, and lipids, into aroma precursors, and to further convert them into a variety of aroma compounds (Thierry et al., 2015). It has been proven that sauerkraut has many benefits for human health, such as promoting digestion, lowering the cholesterol level, and improving the intestinal function (Rabie et al., 2011).

However, up to now, sauerkraut is usually homemade in northeast China and relies on spontaneous fermentation, making it difficult to the production of uniform and high-quality products. During spontaneous fermentation, the microbial community will be altered by various factors, such as the kinds of raw material, the parameters of fermentation process, and the conditions of geographical and climatic (Cao et al., 2017; Li et al., 2017). Therefore, it is essential to investigate microbial community characteristics to identify the primary and core functional microorganisms for improving fermentation management and exploring microbial resources as starter cultures to improve the safety and quality of sauerkraut. Additionally, the correlation between the microbiota and volatile metabolites, and which genus boosts the accumulation of the major flavor metabolites during the fermentation of northeast sauerkraut, is still unclear.

In this study, the microbial composition and succession was characterized by high-throughput sequencing (HTS) technology, and volatilome profile was analyzed via headspace solid-phase microextraction-gas chromatography-mass spectrometry (HS-SPME/GC-MS). Meanwhile, the physicochemical properties and non-volatile metabolites were detected. Furthermore, the correlation between the microbiota and volatile metabolites was explored. This study could provide scientific basis for optimizing and standardizing safety and flavor characteristics of northeast sauerkraut.

## Materials and Methods

### Sample Collection and Preparation

All samples were collected from three different households located in Dalian, Liaoning Province, China. Sampling was carried out according to the process described by Yang et al. (2020). The samples were collected using sterile transfer pipes from the pickle jar at three different sites: the top, middle, and bottom. The five obtained samples from each site (150 mL) were mixed evenly and packed in sterile self-sealing bags. Seventy-two samples from three different households were collected at days 0, 3, 5, 7, 12, 17, 23, 30 and then transported to the laboratory for further analysis.

### Physicochemical Analysis

The pH value was measured by a pH meter (PHS-3G, INESA Scientific Instrument Co., Ltd., Shanghai, China). Titratable acidity (TA) was measured and expressed as lactic acid according to the method described by Yang et al. (2019). The nitrite concentration was measured using hydrochloride naphthodiamide by national standard of China GB 5009.33-2016 (Mao et al., 2018).

### DNA Extraction and PCR Amplification

Microbial community genomic DNA from different samples was extracted using an E.Z.N.A.^®^soil DNA kit (Omega Bio-tek, Norcross, GA, United States) according to manufacturer’s instructions. The integrity of DNA was analyzed on 1% agarose gel electrophoresis, and DNA concentration and purity were determined with NanoDrop 2000 UV-vis spectrophotometer (Thermo Fisher Scientific, Wilmington, DE, United States). Primers pairs 515F (5′-GTGYCAGCMGCCGCGGTAA-3′) and 806R (5′-GGACTACNVGGGTWTCTAA-3′) were used to amplify the hypervariable region V3–V4 of the bacterial 16S rRNA gene by an ABI GeneAmp^®^ 9700 PCR thermocycler (ABI, CA, United States; Walters et al., 2016). PCR amplification conditions were as follows: pre-PCR for 3 min at 95°C, 27 cycles of denaturation at 95°C for 30 s, annealing at 55°C for 30 s, elongation at 72°C for 10 min, and final extension at 72°C for 10 min. PCR amplification was performed in a mixtures, which contained 4 μL buffer (5 × TransStart FastPfu), 0.4 μL DNA Polymerase (TransStart FastPfu), 2 μL dNTPs (2.5 mM), 0.8 μL primer (5 μM), 10 ng genome DNA, and finally made up to 20 μL with ddH_2_O. The amplified DNA was detected by 2% agarose gel followed by purification with the AxyPrep DNA Gel Extraction Kit (Axygen Biosciences, Inc., Union City, CA, United States) according to the manufacturer’s instructions and quantification using the Quantus^TM^ Fluorometer (Promega, United States).

### Illumina Miseq Sequencing and Processing of Sequencing Data

Purified amplicons were pooled in equimolar and paired-end sequenced (2 × 300) using Illumina MiSeq sequencing (Illumina, Inc., San Diego, CA, United States) at Majorbio Bio-Pharm Technology, Co., Ltd (Shanghai, China).

All raw sequences containing an average quality score of <20 and reads shorter than 300 bp were discarded for quality control by Trimmomatic, and merged by FLASH.

The Quantitative Insights Into Microbial Ecology (QIIME, version 1.8.0) was used for the bioinformatics analysis of sequences. According to UPARSE (version 7.1^1^), sequences with similarity more than 97% were clustered and regarded as an operational taxonomic unit (OTU). The RDP classifier^2^ was used to analyze the taxonomy of each OTU representative sequence, against the 16S rRNA database (e.g., Silva SSU128) with confidence threshold of 0.7. The Alpha diversity (including Shannon, Simpson and Coverage) and Beta diversity [principal component analysis (PCA)] analysis were calculated using QIIME software (Version 1.8.0).

### Metabolites Profile Analysis

Metabolites profile analysis, including glucose, fructose, lactic acid, acetic acid, free amino acids (FAAs) and volatile compounds, was performed in triplicate. High-performance liquid chromatography (HPLC) with a differential refractometer CTO-10vp detector (Agilent Instruments, United States) was applied for determination of the concentrations of glucose, fructose, lactic acid and acetic acid at a flow rate of 0.6 mL/min, as previously described by Yang et al. (2019). Briefly, samples were centrifuged at 12,000 × *g* for 10 min and then the supernatant was filtrated through 0.22 μm membrane (Jin Teng Corp., Tianjin, China) before determination. The separation was performed on an Aminex 300 mm × 7.8 mm HPX-87H column (Bio-Rad Laboratories, United States) at 65°C. The mobile phase was 0.005 M H_2_SO_4_. These metabolites were identified and quantified by comparing their retention time and peak areas with standards. FAAs were identified according to the method of Wu et al. (2015). The proteins and peptides in sauerkraut were precipitated by the addition of 10% sulfosalicylic acid and centrifuged at 12,000 × *g* for 20 min. One milliliter of supernatant was filtered through a 0.22 μm micropore filter and evaluated in a High-Speed Amino Acid Analyzer (Biochrom, Ltd., Cambridge, United Kingdom) with a Hitachi 2622 c exchange column (4.6 mm × 6.0 m). FAAs were detected by an ultraviolet detector at wavelengths of 440 nm (Pro) and 570 nm (all the other FAAs). External standard method was used to calculate the quantity of each FAA.

Volatile compounds were determined via HS-SPME/GC-MS as described by Yang et al. (2020). Briefly, 5 mL of the sauerkraut brine and 1.6 g NaCl were transferred to a 15 mL headspace vial. The vial was then tightly capped and heated at 60°C for 40 min in a water bath, stirring every 5 min. Then a 50/30 μm divinylbenzene/carboxen/polydimethylsiloxane SPME fiber (DVB/CAR/PDMS, Supelco, Inc., Bellefonte, PA, United States) was inserted into the vial for the volatile compounds extraction at 60°C water bath for 40 min. GC/MS analyses were performed on a Shimadzu gas chromatograph (Shimadzu, Kyoto, Japan). Separation of volatile compounds was performed on a Rxi-5Sil MS column (30 m length × 250 μm diameter × 0.25 μm thickness, Restek Corporation, Bellefonte, PA, United States). Helium was used as a carrier gas at a constant flow rate of 1.0 mL/min. Oven temperature was maintained at 35°C for 3 min, increased at 6°C/min to 160°C, thereafter increased at 10°C/min to 250°C. The temperature of interface and ion source was set at 230 and 200°C, respectively. The mass spectrometer was operated in electron impact mode with the electron energy set at 70 eV and a scan range of 35–500 m/z. Additionally, 1 μL of 2-octanol in methanol (final concentration of 19.656 μg/L) was used as an internal standard. The volatile compounds identification was conducted using the National Institute of Standards and Technology (NIST) and Wiley libraries (similarity index >85%), followed by quantitative analysis by interpolation of the relative areas versus the area of the internal standard.

### Statistical Analysis

Statistical differences were evaluated by Turkey’s test in SPSS software with *p* < 0.05 considered to denote statistical significance. The line chart and bar chart were generated using Origin Software 8.0. For the data of volatile metabolites, PCA was evaluated by SIMCA 14.0 (Umetrics AB, Umea, Sweden). The correlation between the microbiota and volatile metabolites was estimated using Spearman’s correlation analysis by R 3.3.2 software (Soto Del Rio et al., 2017; Chen et al., 2019). The Benjamini–Hochberg method was used to adjust the *p-*values by false discovery rate (FDR) with FDR < 0.05 considered to denote significant correlations (Benjamini and Hochberg, 1995).

## Results

### Analysis of Physicochemical Characteristics

**FIGURE 1:**
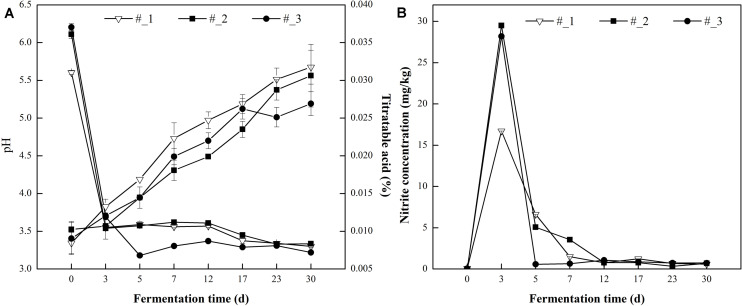
Changes in pH and Titratable acidity (TA) **(A)** and nitrite concentration **(B)** during traditional northeast sauerkraut fermentation from different households. #1, #2, and #3 represented three different households.

The pH value of all samples initially dropped rapidly and stabilized in the subsequent days (Figure 1A). Compared with #1 and #2 samples, the pH displayed the quickest decline in #3 samples. The pH reached a value of 3.30 at day 7 in #3 sauerkraut samples, while in #1 and #2 sauerkraut samples at day 23. At the end of fermentation, no significant difference was found in the pH value among all samples (range from 3.22 to 3.34). The opposite trend was observed in the change of TA which increased throughout the fermentation period and no significant difference was detected among different samples.

As shown in Figure 1B, the nitrite concentration increased to the peak value at day 3, and the maximum level (29.50 mg/kg) was found in #2 sauerkraut. After that, the nitrite concentration dropped to a level below the official maximum limited value (20 mg/kg) and stabilized at an extremely low level (range from 0.58 to 0.73 mg/kg) at the end of fermentation (Li et al., 2017).

### Microbial Composition and Succession

Using HTS technology, a dataset containing 1,030,549 high-quality and classifiable 16S rRNA gene sequences was generated, with an average of 49,074 sequences per sample (range from 38,782 to 59,473). The sequences were classified into 4,617 OTUs at a 97% similarity level, with an average of 220 OTUs per sample (range from 76 to 1157). The Good’s coverage in all samples was 100%, indicating that the quantity and quality of the sequences was sufficient to reveal most microbial communities of samples (Peng et al., 2018).

The alpha diversities, measured via the Shannon and Simpson indices to evaluate richness and diversity of microbial community, were calculated using the QIIME platform (Table 1). The Shannon and Simpson indices are estimators of sampling diversity, and the higher the Shannon index demonstrates the higher the microbial diversity, which is contrary to Simpson index (Qiu et al., 2018). In this study, all samples had the highest bacterial diversity at the beginning of fermentation, which gradually decreased as the fermentation progressed, reaching the lowest level at day 3 in #2 sauerkraut. Thereafter, the microbial diversity increased gradually and the highest level occurred at day 30 in #3 sauerkraut.

**TABLE 1:** Sequence numbers and alpha diversity index values during traditional northeast sauerkraut fermentation from different households.

**Samples**	**Total reads**	**Valid reads**	**OTUs**	**Shannon**	**Simpson**	**Coverage**	**Samples**	**Total reads**	**Valid reads**	**OTUs**	**Shannon**	**Simpson**	**Coverage**
#1_0	52028	49924	361	3.11	0.13	1.00	#2_17	46854	42500	117	2.08	0.28	1.00
#1_3	44739	41410	86	1.32	0.43	1.00	#2_23	47231	40892	118	2.36	0.20	1.00
#1_7	49889	44427	97	1.67	0.33	1.00	#2_30	46852	40544	110	2.29	0.21	1.00
#1_12	49566	44071	104	1.91	0.28	1.00	#3_0	59473	50681	1157	5.07	0.03	1.00
#1_17	52624	46592	106	1.94	0.28	1.00	#3_3	40570	37623	98	1.34	0.43	1.00
#1_23	51825	44073	108	2.23	0.21	1.00	#3_7	45646	41906	118	1.45	0.53	1.00
#1_30	53535	46466	110	2.24	0.21	1.00	#3_12	51362	44939	123	1.95	0.34	1.00
#2_0	38782	33115	1145	5.07	0.03	1.00	#3_17	52618	45590	136	2.22	0.26	1.00
#2_3	42296	39488	76	1.04	0.58	1.00	#3_23	55683	48142	124	2.31	0.22	1.00
#2_7	48395	43733	108	1.71	0.37	1.00	#3_30	57538	48975	117	2.39	0.20	1.00
#2_12	43043	39398	98	1.53	0.45	1.00							

*#1, #2, and #3 represented three different households. The number behind “−” (0, 3, 7, 12, 17, 23, 30) represented fermentation time (day). OTU, operational taxonomic unit.*

**FIGURE 2:**
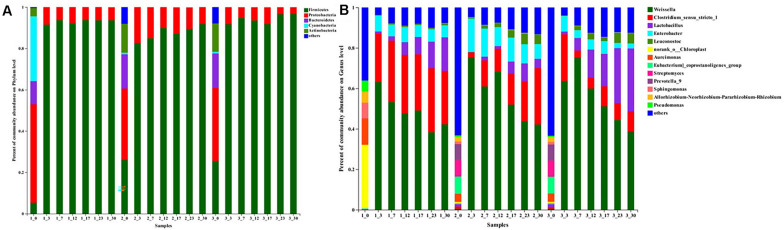
Relative abundance (%) of microbial at the phylum **(A)** and genus **(B)** level during traditional northeast sauerkraut fermentation from different households. #1, #2, and #3 represented three different households. The number behind “−” (0, 3, 7, 12, 17, 23, 30) represented fermentation time (day).

Microbial community at phylum (Figure 2A) and genus level (Figure 2B) in different samples were analyzed. Judging from classification level of phylum, Firmicutes, Proteobacteria, Bacteroidetes, Cyanobacteria, and Actinobacteria were present as major phyla during the early fermentation stage, certain other phyla were also detected but with low relative abundance. At day 0, there was no significant difference in the relative abundance of these phyla between #2 and #3 samples, but differed from that in #1 samples. In detail, Proteobacteria (47.73%), Cyanobacteria (31.38%), Bacteroidetes (11.08%), Firmicutes (5.35%), and Actinobacteria (3.99%) occupied the main position in the #1 samples, whereas Proteobacteria (34.55 and 35.57%), Firmicutes (26.12 and 25.40%), Bacteroidetes (16.49 and 16.59%), Actinobacteria (13.95 and 13.72%) and Cyanobacteria (0.82 and 0.81%) were the predominant phyla in #2 and #3 samples. Subsequently, there was a dramatic increase in the relative abundance of Firmicutes during the first 3 days of fermentation, while the other phyla were present in minor percentage. At the end of fermentation, Firmicutes was the most dominant phylum (range from 91.87 to 96.73%), followed by Proteobacteria (range from 3.16 to 8.09%). Especially in the #3 sauerkraut, Firmicutes rapidly increased to 91.77% within the first 3 days and then gradually increased to the highest value (96.73%) at the end of fermentation, which was significantly higher (*p* < 0.05) than that in the other homemade sauerkraut (93.68 and 91.87%). Other phyla, including Bacteroidetes, Actinobacteria, and Cyanobacteria, declined dramatically throughout the fermentation process and were not detected at the end of fermentation.

At the genus level, there was a significant difference (*p* < 0.05) in the most dominant genera between #1 samples and the other samples at day 0 (Figure 2B). The abundant genera (with average relative abundance of >1%) present in #1 samples were *Chloroplast*, *Aureimonas*, *Sphingomonas*, *Rhizobium*, and *Pseudomonas*, while that in #2 and #3 samples were *Eubacterium*, *Streptomyces*, *Prevotella*, *Aureimonas*, *Rhizobium*, *Sphingomonas*, *Lactobacillus*, and *Clostridium*. Subsequently, *Weissella* was absolutely dominant until the fermentation was completed in all samples. The relative abundance of *Weissella* increased quickly at first and then decreased until the fermentation finished, with the peak value appearing at day 3 in #1 (63.10%) and #2 samples (75.26%) but at day 7 in #3 samples (75.20%), which correlated well with the change of pH and TA (Figure 1A). The second most abundant genus was *Clostridium* in #1 and #2 samples, while it was *Lactobacillus* in #3 samples. At the end of the ripening process, the relative abundance of *Lactobacillus* in #2 samples (4.45%) was significantly (*p* < 0.05) lower than that in #1 (16.68%) and #3 samples (31.12%). At day 30, the relative abundances of *Clostridium* and *Enterobacter* in #2 samples were apparently higher than that in the other samples. In addition, the relative abundance of the genus *Leuconostoc* in #2 (4.71%) and #3 samples (5.13%) was similar at the end of fermentation, but significantly higher (*p* < 0.05) than that in #1 samples (0.96%).

### Multivariate Analysis of the Microbial Community

**FIGURE 3:**
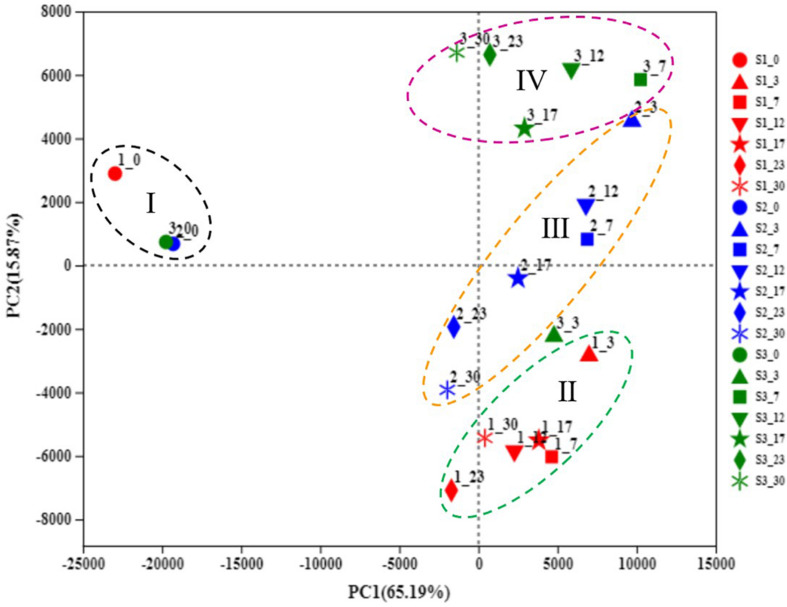
Principal component analysis (PCA) of microbial communities during traditional northeast sauerkraut fermentation from different households. S1, S2, and S3 represented three different households. The number behind “−” (0, 3, 7, 12, 17, 23, 30) represented fermentation time (day). Group I represented all samples at day 0. Groups II, III, and IV represented samples at days 3, 7, 12, 17, 23, 30 from #1, #2, and #3 household, respectively.

Principal component analysis was performed to compare the difference in the microbial community during the fermentation of traditional northeast sauerkraut from different households (Figure 3). The PCA plot (PC1 variance = 65.19%, PC2 variance = 15.87%) showed the scattergram of samples with different fermentation time, according to the OTU level, four groups were automatically clustered. The sauerkraut samples at day 0 were clustered together and lay on the second quadrant (I), particularly for #2 and #3 samples, with the highest degree of similarity. As the fermentation progressed, the discrimination of samples during the mid-late stage of fermentation revealed a trend of moving from top to bottom along PC2 dimension (#3, #2, and #1). Different samples from day 7 to 30 were separated by PC2 and clustered three groups (II, III, and IV). The result showed that the microbial community in traditional northeast sauerkraut changed over time and was distinctly different among sauerkraut samples from different households.

### Target Analysis by HPLC

**FIGURE 4:**
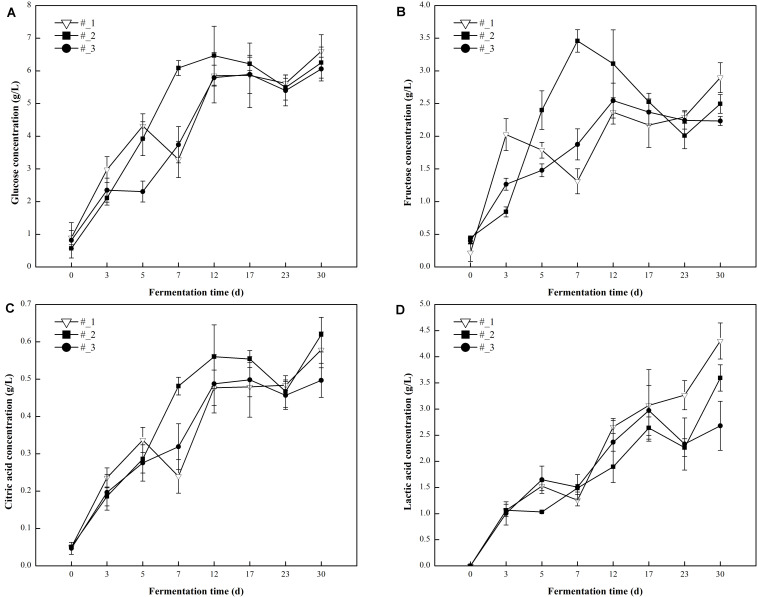
Changes in the concentration of non-volatile metabolites [**(A)**-Glucose, **(B)**-Fructose, **(C)**-Critic acid, **(D)**-Lactic acid] during traditional northeast sauerkraut fermentation from different households. #1, #2, and #3 represented three different households.

Probiotics can catabolize reducing sugars via fermentation leading to the formation of organic acids which are important secondary carbon sources for numerous microorganisms that proliferate during fermentation. In this study, reducing sugars (glucose and fructose) and organic acids (citric acid and lactic acid) were measured during the fermentation of traditional northeast sauerkraut (Figure 4). Within the first 23 days of fermentation, glucose, fructose, citric acid, and lactic acid content increased at first and then decreased in all samples. Subsequently, their contents showed an increased trend which may be related to secondary fermentation by yeast (Franco and Perez-Diaz, 2012). After 30 days of fermentation, the #3 sauerkraut had the lowest concentration of sugars and acids detected in this study. Except for the significantly (*p* < 0.05) higher lactic acid content in #1 sauerkraut samples, there were no significant differences in the other compounds among all samples.

### FAAs Analysis

**FIGURE 5:**
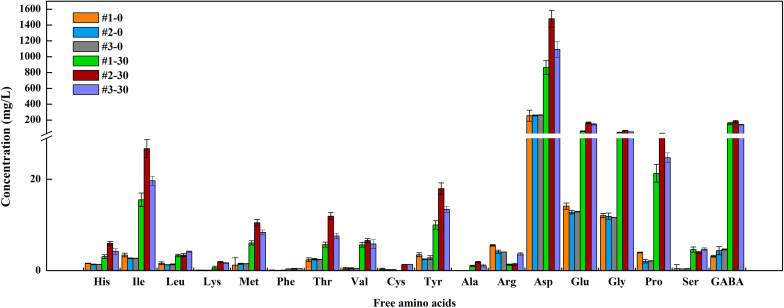
The concentration of free amino acids (FAAs) in different homemade northeast sauerkraut at days 0 and 30. #1, #2, and #3 represented three different households. The number behind “−” (0, 30) represented fermentation time (day).

Free amino acids are the main contributors to the delicate flavor of fermented vegetables. At the end of fermentation, Total FAAs content in #2 sauerkraut was 4- and 7-folds higher than that in #1 and #3 sauerkraut (Figure 5), whereas no significant difference was detected between #1 and #3 sauerkraut (Supplementary Table S2). We observed significant higher content in regard to most of FAAs in #2 sauerkraut in comparison to the other sauerkraut, except for Cys, Leu, Arg, and Ser. Among the 18 detected FAAs, Asp was the most abundant in all samples (71.98, 73.35, and 71.23%), followed by GABA (13.07, 9.02, and 9.20%), and Glu (4.82, 8.15, and 9.61%). The GABA content in ripened sauerkraut from different households increased by almost 50-, 42-, and 30-folds (#1, #2, and #3) as compared to that in initial samples. The Glu (umami taste) content in #2 sauerkraut was three times higher than that in #1 sauerkraut, while was not significantly different from that in #1 sauerkraut.

### Volatilome Profile Determination by HS-SPME/GC-MS

**FIGURE 6:**
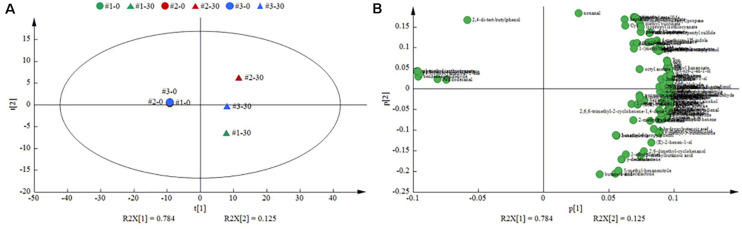
Principal component analysis (PCA) of the volatile metabolites in the traditional northeast sauerkraut from different households. **(A)** Score plot investigated the differences in volatile metabolites among different homemade sauerkraut samples. **(B)** Loading plot identified the volatile metabolites responsible for the separation in score plot. #1, #2, and #3 represented three different households. The number behind “−” (0, 30) represented fermentation time (day).

A total of 130 volatile compounds were detected, including 11 acids, 16 alcohols, 24 esters, 22 aldehydes, 3 ketones, 6 nitriles, 5 isothiocyanates (ITCs), 7 sulfides, 2 indoles, 5 hydrocarbons, 7 phenols, 6 lactones, and 16 terpenes (Supplementary Table S1). In order to better understand the differences among different sauerkraut samples from three households, a PCA of the 130 volatile compounds recorded at days 0 and 30 in all sauerkraut samples was calculated (Figure 6A). The first and second principal components (PCs) showed that the cumulative percentage variance of volatiles accounted for 78.4 and 12.5%, respectively. Significant change in volatile profile was found between the beginning (day 0) and finished time points (day 30), which revealed a trend of moving from left to right along PC1 dimension as the fermentation progressed. Regarding the loading plot (Figure 6B), the #1 sauerkraut was distinguished by the highest contents in acids (acetic acid, butanoic acid, hexanoic acid, and 3-methylbutanoic acid), lactones (γ-dodecalactone, δ-nonalactone, and γ-decalactone), dihydrocarveol, 1-butanol, hexanal, and methanethiol. The #2 sauerkraut was characterized by the highest contents in alcohols (ethanol and 1-hexanol), aldehydes (octanal, nonanal, and benzaldehyde), esters (ethyl butanoate, ethyl acetate, hexyl acetate, isoamyl acetate, and phenethyl acetate), sulfides (dimethyl disulfide and dimethyl trisulfide), ITCs, and phenols. However, the #3 sauerkraut was featured by the highest contents in terpenes [α-terpineol and (Z)-3,7-dimethyl-2,6-octadienol], octanoic acid, isoamyl alcohol, decanal, ethyl octanoate, ethyl lactate, ethyl isovalerate, and nitriles.

The total content of acids in #1 sauerkraut (30.53 ng/L) was significantly (*p* < 0.05) higher than that in the other sauerkraut samples (16.48 and 16.82 ng/L), especially acetic acid (sour taste and smell), butanoic acid (rancid butter and spicy taste), hexanoic acid (yogurt and floral flavor), and 3-methylbutanoic acid (rancid and sweaty odor) (Costa et al., 2019). In addition, level of total alcohols was found in significantly greater amount in #2 sauerkraut than that in the other sauerkraut, with the predominant being ethanol, 1-hexanol (fruity aroma), and phenylethyl alcohol (rose and honey-like odor) (Jin et al., 2019).

Compared with the other sauerkraut samples, the content of total esters in #2 sauerkraut samples was significantly higher. Ethyl lactate and ethyl isovalerate were detected only in #3 sauerkraut, which were recognized as being fruity and floral aroma with lower detection threshold (Ly et al., 2019). Similarly, lactones also have fruity and floral odor notes, which contribute significantly to the flavor characteristic of fermented foods (Lee et al., 2018). The content and type of lactones showed significant differences among all samples, with the highest level in #1 sauerkraut.

The total sulfides content in #2 sauerkraut (12.68 ng/L) was four and three times higher than that in #1 (3.94 ng/L) and #3 sauerkraut (2.70 ng/L), respectively. Especially, dimethyl disulfide and dimethyl trisulfide were the most abundant sulfur-containing compounds, which contributed a cabbage-like odor and had been identified as the marked compounds in sauerkraut. ITCs and nitriles are known to be unique flavor of *Brassica* vegetables, which are produced through the degradation of glucosinolates by the catalytic activity of myrosinase (Tomita et al., 2018). The content of these compounds was significantly higher in #2 sauerkraut than that in the other sauerkraut.

Compared with #3 sauerkraut, the #1 and #2 sauerkraut showed a higher amount of total aldehydes, represented by hexanal, which imparted a green odor, showed antimicrobial activity, and protected plants from pathogens (Jampaphaeng et al., 2018). Although ketones were commonly found in the majority of fermented vegetables, in this study, they were present in low amount (range from 1.84 to 2.46%).

### Correlation Analysis Between Microbiota and Volatile Metabolites

**FIGURE 7:**
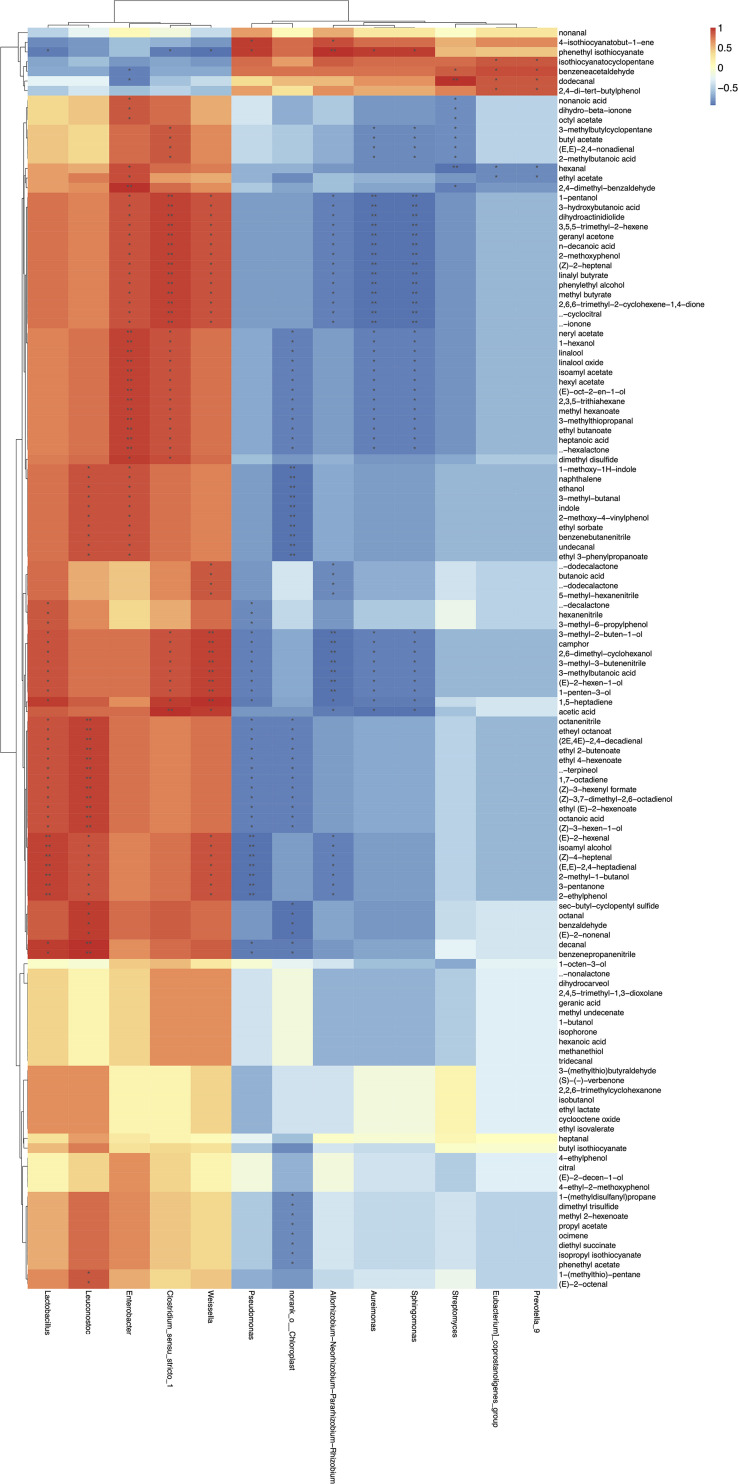
Heatmap of the correlations between microbiota and volatile metabolites. The red color represented positive correlation and the blue color represented negative correlation. “*” represented the significance of relationship, “*” represented 0.01 < *p* ≤ 0.05, “**” represented 0.001 < *p* ≤ 0.01.

Finally, in order to determine the correlations among the microbial community and volatile compounds in homemade northeast sauerkraut, correlation matrixes were established based on spearman correlation coeffcients (Figure 7). Results showed that different microorganisms contributed differently to the volatile flavors (—*r*— > 0.7, *p* < 0.05). The main bacteria during fermentation of northeast sauerkraut, that were, *Pseudomonas*, *Chloroplast*, *Rhizobium*, *Aureimonas*, *Sphingomonas*, *Lactobacillus*, *Leuconostoc*, *Enterobacter*, *Clostridium*, and *Weissella*, were statistically correlated with the volatile compounds. As highlighted, several negative correlations were found between microbiota and volatile compounds. In detail, *Pseudomonas* showed a negative correlation with 33 kinds of volatile compounds, including nitriles (benzenepropanenitrile and octanenitrile), phenethyl isothiocyanate, octanoic acid, α-terpineol, γ-decalactone, isoamyl alcohol, decanal, ethyl octanoate, etc. *Chloroplast* exhibited strong negative correlation with 49 kinds of volatile compounds, including aldehydes (octanal, decanal, and benzaldehyde), esters (ethyl butanoate, isoamyl acetate, and phenethyl acetate), nitriles (benzenebutanenitrile and octanenitrile), heptanoic acid, linalool, δ-hexalactone, ethanol, dimethyl trisulfide, isopropyl isothiocyanate, etc. *Rhizobium* was negatively correlated with 36 kinds of volatile compounds, including lactones (γ-dodecalactone and δ-dodecalactone), aldehydes [(Z)-4-heptenal and (E, E)-2,4-heptadienal], β-ionone, isoamyl alcohol, etc. *Aureimonas* showed a significant negative correlation with acids (acetic acid and 3-methylbutanoic acid), alcohols (1-hexanol and phenylethyl alcohol), esters (ethyl butanoate and isoamyl acetate), linalool, δ-hexalactone, and 3-methyl-3-butenenitrile; the same correlation was seen in the genus of *Sphingomonas*. On the contrary, *Clostridium*, *Enterobacter*, *Lactobacillus*, *Leuconostoc*, and *Weissella* were the main contributors to the production of volatile flavors of sauerkraut. *Lactobacillus* exhibited a significant positive correlation with 33 kinds of volatile compounds, which mainly include acids (octanoic acid and 3-methylbutanoic acid), aldehydes [decanal, (Z)-4-heptenal and (2E, 4E)-2,4-decadienal] terpenes [α-terpineol and (Z)-3,7-dimethyl-2,6-octadienol], nitriles (benzenepropanenitrile, hexanenitrile, and octanenitrile), isoamyl alcohol, ethyl octanoate, etc. *Leuconostoc* positively correlated with 37 kinds of volatile compounds, including alcohols (ethanol and isoamyl alcohol), aldehydes (octanal, decanal, benzaldehyde, and 3-methyl-butanal), nitriles (benzenebutanenitrile and octanenitrile), etc. *Enterobacter* and *Clostridium* displayed the same situation of a positive correlation with terpenes (β-ionone, linalool), alcohols (1-hexanol and phenylethyl alcohol), esters (ethyl butanoate and isoamyl acetate), δ-hexalactone, heptanoic acid, dimethyl disulfide, etc. The absolute predominant genus *Weissella* positively correlated with 35 kinds of volatile compounds, including acids (acetic acid, butanoic acid, and 3-methylbutanoic acid), terpenes (β-ionone and β-cyclocitral), lactones (γ-dodecalactone and δ-dodecalactone), alcohols (phenylethyl alcohol, isoamyl alcohol, and 1-pentanol), aldehydes [(Z)-4-heptenal and (E,E)-2,4-heptadienal], etc. In addition, several genera, including *Streptomyces*, *Eubacterium*, and *Prevotella*, showed weaker correlations with volatile compounds.

## Discussion

In this study, a comprehensive investigation of the physicochemical property and microbial community, as well as the metabolome profile in traditional homemade northeast sauerkraut was carried out. What’s more, the correlation between microbiota and volatile metabolites was established using statistical methods and bioinformatics tools.

Regarding the physicochemical characteristics, pH is a key factor driving the change in microbial community structure during sauerkraut fermentation, which is used to assess the maturity of fermented vegetables (Li et al., 2017). In this study, the changing trend of pH was similar to those of other fermented vegetables in previous studies (Xiong et al., 2016; He G. et al., 2020) which showed a rapid decline initially and then remained stable. In addition, nitrite is the key factor affecting the safety of sauerkraut, which is transformed from nitrate rich in cabbages and can induce methemoglobinemia or cancer. As the fermentation proceeded, LAB became the dominant bacteria which resulted in a higher acid environment, accelerated the degradation of nitrite to nitric oxide and inhibited the growth of nitrate-reducing bacteria (Zhang et al., 2019). This is likely to have contributed to the subsequent rapid decrease in nitrite concentration to a level far lower than the official maximum limited value (20 mg/kg).

HTS technology was used to investigate the microbial diversity and succession during fermentation of homemade northeast sauerkraut. In all samples, the microbial diversity decreased gradually during the first 3 days of fermentation, which was analogous to the change of pH value. The initial microorganisms observed at day 0 were not only from the cabbage itself, but possibly also from the environment such as floor, tools and air, and could be introduced during the process of the material pretreatment process. The result indicated that due to the accumulation of acids, the more exogenous microorganisms which were not tolerant to a higher acidic environment were temporarily inhibited. However, when microbial communities had adapted to the acidic and salty environmental condition, the Shannon index showed an increasing trend until the end of the fermentation (Table 1). Therefore, as the fermentation progressed, the microbial community shifted sequentially from less acidic tolerant group to more acidic tolerant one that were more adaptable to the acidic and salty environmental condition (Liang et al., 2018). The result confirmed that the microbial diversity relied not only on the fermentation process but also the fermentation environment (Zhu et al., 2018). The dramatic decrease in the phylum Proteobacteria was a result of spontaneous fermentation and a previous study had also reported a decrease in Proteobacteria and an increase in Firmicutes during kimchi fermentation (Kang et al., 2018). *Weissella* was dominant in all samples throughout the entire process, which was inconsistent with some previous reports on other fermented vegetables in which *Leuconostoc* was dominant at the beginning of fermentation and *Lactobacillus* dominated in the late stage of fermentation (Cao et al., 2017; Guan et al., 2020). The difference is significantly based on the material diversity, geographical distribution, and production process. The predominance of *Weissella* throughout the entire fermentation period could be explained by the hypotheses that *Weissella* is more competitive under acidic condition (Jung et al., 2012). In addition, *Clostridium* was detected in higher relative abundance at the end of fermentation, and similar result was reported by Zhou et al. (2018).

The relative abundance of *Lactobacillus* increased in conjunction with the decrease in the relative abundance of *Rhizobium*, *Aureimonas*, *Enterobacter*, and *Pseudomonas*, especially in the #3 sauerkraut. A probable explanation is that *Lactobacillus* can produce lactic acid and a variety of antimicrobial substances such as bacteriocins that can inhibit the growth of pathogens and spoilage microorganisms during the fermentation process (Huang et al., 2019). In addition, *Clostridium* and *Enterobacter* were generally considered to be spoilage bacteria in fermented cucumber pickles (Franco and Perez-Diaz, 2012) which were apparently higher in #2 samples than that in the other samples at day 30. The existence of *Enterobacter* is undesirable due to the competition with LAB for available nutrients and production of nitrite (Nazar et al., 2020). However, in recent years, there were many reports about the food-borne pathogenic bacteria in acidic foods, which deserved our close attention (Wang et al., 2020). *Pseudomonas*, *Sphingomonas*, and *Rhizobium* are widely present in the environment and can attach to the surface of raw materials, which might relate to poor sanitary condition and easily lead to the spoilage of fermented vegetables such as tomatoes, cabbages, corn lettuce, and cucumbers (Pérez-Díaz et al., 2019). Previous studies have reported that there are the risk of microbial safety and potential of deterioration in some homemade fermented vegetables, suggesting that these products might be contaminated during the production process due to the unhygienic production environment (Cao et al., 2017; Guan et al., 2020). Hence, it is necessary to take appropriate measures to prevent the contamination caused by opportunistic pathogens and other microbial. It is well established that the microbiota during fermentation of sauerkraut originate from raw materials, environment and production area. In this study, the microbial community exhibited significant differences, which may be put down to the differences in the raw material composition and environmental microorganisms.

Different microbial community during fermentation could lead to the difference in the metabolome profile. During sauerkraut fermentation, the changes of reducing sugars and organic acids are important indicators which indirectly reflect the activities of microorganisms. The dominant carbohydrates of cabbage (glucose and fructose) still remained at the end of fermentation in all samples, indicating the inadequate consumption of these compounds during the fermentation of homemade northeast sauerkraut. In line with a previous report showing that *Leuconostoc* was able to use fructose as alternative external electron acceptor to produce mannitol (Harth et al., 2016) the lowest concentration of fructose and the higher relative abundance of *Leuconostoc* were observed in #3 sauerkraut at day 30. The types of organic acids produced by the homolactic and heterolactic fermentation with LAB are different (Liang et al., 2018). The predominant organic acid in this study was lactic acid, while citric acid concentration remained at low level. The release of lactic acid by LAB not only reduces the pH and inhibits the spoilage microorganisms, but also has a positive effect on the organoleptic characteristics of sauerkraut through esterification with alcohols which reduces the irritating taste and provides an enhanced aroma (Xu et al., 2019). This might account for the lowest concentration of lactic acid, the highest content of ethyl lactate, and the lowest relative abundance of spoilage bacteria in #3 sauerkraut.

Together with acidification, proteolysis is considered a key feature in food biotechnology, since the degradation of native proteins is of great importance for improving the digestibility of polypeptides and bioavailability of FAAs, but also for the release of potential bioactive peptides and for the development of taste and flavor (Rizzello et al., 2018). FAAs catabolism leads to a wide of flavor compounds, such as aldehydes, alcohols, esters, acids, and sulfur compounds (Xiao et al., 2019). In this study, we observed an increase in the total FAAs content among all samples, which was consistent with the results of previous studies on other fermented vegetables (Wu et al., 2015; Rao et al., 2020). FAAs and their degradation products have an impact on the quality of fermented foods, such as sensory property (umami taste of Glu and Asp, sweet taste of Ala and Thr) and a beneficial effect associated with GABA (Zhao et al., 2016). In this study, Glu and Asp were particularly predominant, which contributed most to the umami of sauerkraut. GABA is an amino acid derivative produced from the irreversible α-decarboxylation of Glu by glutamate decarboxylase in LAB and is widely studied for the hypotensive effect that plays a fundamental role in central nervous system, it is involved in the regulation of blood pressure, heart rate, and alleviation of pain and anxiety (Verni et al., 2019). What’s more, GABA-enriched foods are required because the GABA content in the typical daily human diet is relatively low (Cho et al., 2011). The enhancement of sulfur amino acids (Met and Cys) was mainly attributed to the hydrolysis activity of endogenous and microbial enzymes on myosin and myofibrillar proteins, which made an important contribution to improve the nutrition of products (Du et al., 2019). In #2 sauerkraut samples, the highest concentration of aromatic amino acids (Phe and Tyr) correlated well with the highest content of phenols, which was consistent with the fact that aromatic amino acids can be converted into phenols (Park and Kim, 2019).

Flavor from volatile compounds is one of the most important characteristics of products for consumer palatability. PCA analysis showed that the volatilome profile after fermentation was remarkably different from that at the beginning of fermentation. At the end of fermentation, all sauerkraut samples were clearly distinguished, suggesting that the flavor of sauerkraut from different households was different.

Among volatile acid compounds, hexanoic acid was detected only in #1 sauerkraut, which was considered as a major contributor to the sensory profile of northeast sauerkraut (Wu et al., 2014). The content of butanoic acid in #1 sauerkraut (13.04 ng/L) was higher than that in #3 sauerkraut (1.62 ng/L), whereas no butanoic acid was detected in #2 sauerkraut. The result was in accordance with that reported by Shim et al. (2012). Butanoic acid was the most interesting short-chain fatty acid, which could *in vivo* suppress inflammation to improve nonalcoholic steatohepatitis, and its sodium compound was reported to have influence on the brain metabolism and have potential to modulate sleep (Ye et al., 2018; Szentirmai et al., 2019). Yin et al. (2019) reported that butanoic acid produced by *Lactobacillus plantarum* enhanced the function of coix seed and improved the palatability with an acid flavor. Additionally, it is worth noting that butanoic acid has an unpleasant rancid butter odor and a spicy taste, therefore lower level is desirable (Jin et al., 2019). Higher concentration of acetic acid in #1 sauerkraut may be explained by the mechanism of resistant living cells to overcome lactic acid stress, which can reroute the pathway of acetic acid production through the conversion of pyruvate to acetyl-CoA and acetyl-P by the overexpression of pyruvate metabolic enzymes at the expenses of lactic acid (Nsogning et al., 2018). The highest content of alcohols in #2 sauerkraut may be related to the highest content of aldehydes. According to Lu et al. (2019) aldehydes derive from amino acid metabolism, and convert to corresponding acids and alcohols through oxidation-reduction reaction.

Esters are an important group of sensory-active compounds contributing to fruity and floral flavor of fermented vegetables, and generally originate from esterification of short-chain acids with alcohols. The dominant ester compound identified in this study (ethyl butanoate and strawberry-like aroma) was different from a previous study by Wu et al. (2015) who reported that ethyl acetate (pineapple aroma) and ethyl lactate (fruity aroma) were the dominant esters in suan cai.

Terpenes are the major compounds responsible for floral note, and show antioxidant and antimicrobial activity (Minervini et al., 2020). Compared with #1 and #2 sauerkraut, higher content of terpenes was detected in #3 sauerkraut. The increase of terpenes could be related with the ability of microorganisms to produce glycosylases, which are able to cut the bond between terpenes and sugars, or to their possible *ex novo* production (Ricci et al., 2018).

Aldehydes are considered the key flavor compounds in fermented vegetables with sweet, fruity, nutty, and caramel-like odors, which are produced by unsaturated fatty acids oxidation and amino acids catabolism (Liu et al., 2020). In addition, aldehydes can also be reduced to the corresponding alcohols or oxidized to the corresponding acids (Zhao et al., 2020). A significantly higher content of aldehydes was found in #2 sauerkraut when compared with that in the other sauerkraut, especially, octanal (fruity aroma), nonanal (rose-like odor), 3-methyl-butanal (cheesy and apple-like odor), and benzaldehyde (natural almond odor; Kunjapur and Prather, 2015) indicating that the amino acid metabolism during the fermentation of #2 sauerkraut was active. Moreover, nonanal has been proved to have a significant effect on the antidiarrhoeal (Zavala-Sánchez et al., 2008). So, nonanal plays an important role in the flavor and health care function of the northeast sauerkraut.

Lactic acid bacteria can contribute to the formation of free fatty acids, which can be precursors of lactones and other characteristic aroma compounds in fermented vegetables (Lee et al., 2018). The composition of lactone compounds was quite different among different homemade sauerkraut samples, which may be attributable to the difference in the relative abundance of *Lactobacillus*.

Among different homemade sauerkraut samples, the content of ITCs and nitriles was significantly different. Meanwhile, ITCs and nitriles detected in this study differed from a previous study reported by Wu et al. (2015). This result probably indicated that the level of these compounds depended on various fermentation conditions such as the glucosinolate content of raw plant materials, the activity of myrosinase, the diversity of microbial community, and the level of pH value (Tomita et al., 2018). With respect to glucosinolate-derived ITCs and nitriles, in the salted pickle production of *Brassica* vegetables, it has been reported that they have attracted an extensive attention in terms of the potential to prevent certain types of cancer (Palomino et al., 2015). Sulfides with onion and cabbage aroma have been reported as the major contributor to the characteristic flavor of fermented *Brassica* vegetables, which are considered to be derived from the degradation of sulfur-containing amino acids (Cys and Met) (Cecchi et al., 2020). In this study, dimethyl disulfide (onion, cabbage, and putrid odor) and dimethyl sulfide (cabbage, sulfur and gasoline odor) were the most dominant sulfur compounds, which was agreed with the result of Chen et al. (2013). In addition, the accuracy, specificity and recovery of the conditions for HS-SPME/GC-MS used in this study should be verified in future study.

The correlation between microbiota and volatile metabolites during the traditional fermentation of homemade northeast sauerkraut is still not clear. In this study, spearman’s correlation analysis was used to integrate the microbiota and volatilome dataset in order to dig into the potential correlation between microbiota and volatile compounds in homemade northeast sauerkraut. Eventually, *Clostridium*, *Enterobacter*, *Lactobacillus*, *Leuconostoc*, and *Weissella* appeared to be core flavor-contributing genera for the homemade northeast sauerkraut. Supportive evidences were found in previous studies regarding some of the correlations. *Lactobacillus* showed strong correlation with acids, esters and isoamyl alcohol, which was in line with a previous study on the fermentation of dairy beverage kefir (Walsh et al., 2016). The positive correlation between *Weissella* and lactones was related to the ability of LAB to produce free fatty acids, which were precursors of lactones (Lee et al., 2018). Notably, *Enterobacter* was identified as an important genus related to volatile compounds, being in good agreement with a previous study by He G. et al. (2020). This was the first report that *Clostridium* made great contributions to the flavor formation of sauerkraut, while previous report found that *Clostridium* was an important genus for production of hexanoic acid in the liquor brewing microecosystem (He Z. et al., 2020). The correlation analysis between microbiota and volatile metabolites could facilitate the selection of starter strains and the optimization of fermentation, producing sauerkraut products with better flavor and nutritional value.

This study presented a detailed analysis and comparison of the physicochemical property, microbial diversity and metabolome profile in traditional northeast sauerkraut collected from different households. In addition, the correlation between microbiota and volatile metabolites was analyzed simultaneously and may be useful for the development of starter cultures suitable for northeast sauerkraut production. The result indicated that it was not standardized and there was microbial safety risk and potential for spoilage in homemade northeast sauerkraut products. Thus, future studies should focus on the role of each microbial species, the selection of an optimal inoculant and optimization of the fermentation process. However, it should also be addressed the limitations of this study that the uncontrolled environmental condition of homemade sauerkraut and limited access to collect more samples from other regions. So, future studies are still needed to verify the correlation between microbiota and flavors in traditional northeast sauerkraut.

## Data Availability Statement

The original contributions presented in the study are included in the article/Supplementary Material, further inquiries can be directed to the corresponding author.

## Author Contributions

XZY, WH, ZX, and AJ: conceptualization. WH: methodology, project administration, and funding acquisition. XZY: validation, formal analysis, data curation, and writing – original draft preparation. XZY and XGY: investigation. XZY, WH, ZX, AJ, XGY, GS, YJ, YG, and KF: writing – review and editing. WH and ZX: supervision. All authors contributed to the article and approved the submitted version.

## Conflict of Interest

The authors declare that the research was conducted in the absence of any commercial or financial relationships that could be construed as a potential conflict of interest.

## Footnotes

1 http://drive5.com/uparse/

2 http://rdp.cme.msu.edu/

## Supplementary Material

TABLE S1 The detailed concentration of volatile metabolites in different homemade northeast sauerkraut at days 0 and 30. #1, #2, and #3 represented three different households. The number behind “−” (0, 30) represented fermentation time (day). RT, retention time; Total, the total concentration of each class of volatile metabolites. Mean values in the Total row with different superscripted letters indicate that they are significantly different at *p* < 0.05. ANOVA analysis was performed using Tukey’s test.

TABLE S2 Detailed quantitative information of each free amino acids (FAAs) in different homemade northeast sauerkraut at days 0 and 30. #1, #2, and #3 represented three different households. The number behind “−” (0, 30) represented fermentation time (day). Total, the total concentration of 18 FAAs identified in this study. Mean values in the same row with different superscripted letters indicate that they are significantly different at *p* < 0.05. ANOVA analysis was performed using Tukey’s test.
